# Meat Acquisition Skill and Male Reproductive Success in Guinea Baboons

**DOI:** 10.1002/ajpa.70327

**Published:** 2026-08-05

**Authors:** William J O'Hearn, Federica Dal Pesco, Roger Mundry, Julia Fischer

**Affiliations:** ^1^ Department of Primate Cognition, Georg‐August‐Universität Göttingen Johann‐Friedrich Blumenbach Institute Göttingen Germany; ^2^ Cognitive Ethology Laboratory German Primate Center Göttingen Germany; ^3^ Leibniz ScienceCampus Primate Cognition Göttingen Germany; ^4^ Center for Research in Animal Behaviour University of Exeter Exeter UK

**Keywords:** female partner choice, Guinea baboon, male quality signal, meat sharing, meat transfer, reproductive success

## Abstract

**Objectives:**

In human foraging societies, hunting skill is often interpreted as a signal of male quality linked to reproductive success through his ability to provision his family and community. Similarly, in some bird and insect species, males offer mates food to indicate their quality as a provider. Among nonhuman primates, however, the relationship between meat sharing and reproductive success is underexplored, leaving the evolutionary origins of this phenomenon unresolved.

**Materials and Methods:**

Guinea baboons ( *Papio papio* ) are a promising model to investigate whether meat sharing signals male quality, since females choose their mates, have been shown to rely on males to catch and transfer prey, and respond to male foraging skill. We combined records of 109 meat‐eating events with 9 years of behavioral data to test whether males who more frequently acquired and transferred meat to females had more females in their social units for longer.

**Results:**

Contrary to our predictions, we found no evidence that females preferred males who acquired or transferred meat more frequently, suggesting that meat acquisition is not a signal of male quality in Guinea baboons.

**Discussion:**

One explanation may be the relatively low frequency of meat‐eating events. Another is that females are less dependent on males for meat than previously reported. Our results revealed that nearly half (41%) of female meat intake originated from sources besides her unit male, including the first documented cases of female prey capture (11% of events) in this species. Thus, females likely apply other, more pertinent criteria in mate choice.

## 1 Introduction

In human hunter‐gatherer societies, successfully catching prey can signal a male's quality as a provider, and sharing the spoils of the hunt can broadcast that signal throughout his community (Gurven and Von Rueden 2006; Jaeggi and Gurven 2013, 2018). In such societies, meat is a highly prized food source that often arrives in quantities greater than a single individual or household can consume (Wood and Marlowe 2013). Consequently, the cost of sharing is small, but the benefit of receiving is significant, leading meat to be shared more widely than other foods (Hawkes et al. 1991; Hill et al. 1993; Jaeggi and Gurven 2013; Kaplan and Hill 1985). Men who share more meat more often provide their immediate family with difficult‐to‐acquire nutrients, experience increased reputation within their community, and are sought after as both mates and extra‐pair mating partners (Alvard and Gillespie 2004; Gurven 2004; Gurven and Von Rueden 2006; Hill et al. 1993; Wiessner 1996). Thus, frequent meat providers experience higher reproductive success in most hunter‐gatherer societies (Smith 2004; Wiessner 1996).

While the extent of human meat sharing is unique, the practice of catching and sharing prey as a signal of male quality has deeper evolutionary roots. Males of numerous insect (Vahed 1998) and some arachnid ( *Paratrechalea ornata* , Albo and Costa 2010) and bird species ( *Corvus corax* , Heinrich 1988; *Corvus monedula* , de Kort et al. 2006; *Corvus frugilegus* , Emery et al. 2007; *Sterna hirundo* , González‐Solís et al. 2001; *Netta rufina* , Amat 2000) provide prospective mates with “nuptial gifts” or “courtship feeding” to signal their quality as a provider and encourage mating opportunities, with males that share more or larger prey items generally having better reproductive outcomes. However, within the clade of our closest living relatives, nonhuman primates, in which we might expect to detect the evolutionary origins of human meat‐sharing practices, there are no convincing examples of meat sharing as a “costly signal” of male quality (Jaeggi and Gurven 2013). Nor is there substantial evidence of a relationship between male meat sharing and reproductive success (Jaeggi and Van Schaik 2011).

The bulk of the research on meat sharing has focused on chimpanzees ( *Pan troglodytes* ) as the only taxon that hunts in groups and shares meat actively (Boesch and Boesch 1989; Watts 2020). Studies exploring a possible link between meat sharing and reproductive success in chimpanzees have yielded mixed results. Following a meat‐sharing event, male chimpanzee hunters have been reported to receive immediate mating opportunities (Stanford 1998), delayed mating opportunities (Achorn et al. 2023; Gomes and Boesch 2009), or no notable increases in mating success (Mitani and Watts 2001). The transfer of meat between individuals is not only restricted to apes. Capuchins and baboons are the two other primate taxa in which meat‐eating is most observed and best described (Watts 2020). While neither capuchins (Perry and Rose 1994) nor baboons (Allan et al. 2022; Goffe and Fischer 2016; Harding 1975; O'Hearn, Neumann, et al. 2025; Rhine et al. 1986; Schreier et al. 2019; Strum 1975) perform owner‐initiated “active sharing,” they both transfer meat to others with varying degrees of tolerance up to and including recipient‐initiated “passive sharing”—also termed tolerated theft (Stevens and Gilby 2004). However, it remains uninvestigated whether the frequency with which males provide meat to others functions as a signal of male quality in these species (Jaeggi and Gurven 2013; Watts 2020). Baboons, in particular, offer a valuable analogy for human hunter‐gatherer diet and food practices given their shared ecological history, parallel evolution with early hominids, and, in some species, the similarity of their societies' multilevel social organization (Chapais 2009; Codron et al. 2008; DeVore and Washburn 1963; Fischer et al. 2019; Grueter et al. 2020; Jolly 1970).

In this study, we investigated Guinea baboons ( *Papio papio* ) as a system in which males' ability to acquire and transfer meat may signal male quality to females and thus impact their reproductive success. Guinea baboons live in a multilevel society, at the base of which are stable “units” comprised a reproductive “primary male” and one to several females with their young (Anderson and McGrew 1984; Boese 1975; Dal Pesco et al. 2022; Goffe et al. 2016; Sharman 1982). Units are nested within “parties,” and parties are nested within “gangs” (Fischer et al. 2017; Goffe et al. 2016; Patzelt et al. 2014). At the unit level, male–female tenure is variable—some associations last for weeks, while others last for years (Goffe et al. 2016). Once in a unit, females demonstrate high mate fidelity, with primary males siring 91.7% of offspring in their units, making the number of associated females in a male's unit and female tenure strong indicators of male reproductive output (Dal Pesco et al. 2022).

Guinea baboons appear to be a promising system for investigating whether male meat‐transferring frequency serves as a signal of male quality. Female Guinea baboons exhibit uncoerced choice of mates, often dispersing across multiple levels of the society to join a male's unit (Dal Pesco et al. 2022; Fischer et al. 2017). Curiously, however, the criteria by which females choose males are unclear. Researchers have tested a number of possible criteria for female choice, including preferences for specific secondary sexual characteristics (in prep.) and for males with stronger social connections with other males (Dal Pesco et al. 2022). However, none of these traits have been found to explain variation in the number of females associated with a male nor a female's tenure in his unit. This study extends the search for a female mate choice criterion in Guinea baboons by testing whether females prefer males who catch and transfer meat more frequently—thus provisioning females with key, rare, micronutrients (Tennie et al. 2009; Watts 2020). Moreover, a male's willingness to transfer meat may reflect his general disposition to allow co‐feeding with unit females and offspring. A previous study of meat eating in Guinea baboons revealed that one's access to meat is determined by the relationship quality with the owner (O'Hearn, Beckmann, et al. 2025). An earlier study based on a small number of meat‐eating events reported that male Guinea baboons caught prey, then transferred it preferentially to females in their units, who ate 10%–40% of the meat males acquired (Goffe and Fischer 2016). These findings suggest that female Guinea baboons may depend on their males for access to meat. Finally, in a recent experiment, female Guinea baboons were shown to monitor their males' foraging skills and to compete over access to them when those males were the only individuals that could operate an automated feeder that produced a high‐quality sharable food source (O'Hearn, Beckmann, et al. 2025). This finding indicates that females can detect differences in the type or amount of food their males provide access to and prefer males who can provide more. Thus, female Guinea baboons appear to possess both the means and motivation to inform their choice of mate with input regarding males' abilities to provide them with access to meat. We therefore predicted that if females consider male meat acquisition success and meat transfer frequency as features of their quality, then males who excel at these behaviors should have (1) more associated females and (2) retain females in their units for longer.

## 2 Methods

### 2.1 Field Site and Study Subjects

We carried out the fieldwork for this study from April 2014 to June 2023 using the population of Guinea baboons at the field station “Centre de Recherche de Primatologie (CRP) Simenti” (13°01′34″ N, 13°17′41″ W), in the Niokolo‐Koba National Park, Senegal. Across that period, the study population included 465 individually identified animals of all age and sex classes from both focal gangs, in which all party members were identified, and adjacent parties, in which only a few individuals were known. Baboons in the focal parties were fully habituated and individually identified by natural markings, body shape and size, and radio collars. The population comprised two gangs, totaling 11 parties, which varied in size and composition during the study period. The average number of adults across all parties and years was 4.47 for males (range: 1–13) and 4.50 for females (range: 1–11).

Like other baboons, Guinea baboons are eclectic omnivores (Ohrndorf et al. 2025; Zinner et al. 2021) that occasionally acquire meat from birds or small mammals (Zinner et al. 2021). We use the term “acquire meat” because Guinea baboons opportunistically catch and eat prey they encounter rather than actively “hunting” prey like humans or chimpanzees (Goffe and Fischer 2016; Hawkes et al. 1991; Mitani and Watts 2001; Watts 2020). Their main prey are young bushbucks ( *Tragelaphus scriptus* ), which are too large for a single baboon to consume (young bushbuck: 10–14 kg, adult Guinea baboon: 10–26 kg). Thus, as in human societies, meat from a single prey is usually transferred between and eaten by multiple individuals—up to 11 in a single event (O'Hearn, Beckmann, et al. 2025). Meat can be transferred by individuals picking pieces from the carcass and the surrounding ground (passive sharing) or by taking fallen pieces farther away as the carcass is moved (scavenging). Individuals can also take possession of a carcass once its previous possessor moves off (succeeding) or by aggressively taking the carcass from its possessor (stealing; O'Hearn, Beckmann, et al. 2025).

### 2.2 Female–Male Associations Data Collection

We monitored female–male associations and unit composition in eight focal parties using observational and demographic data collected from April 2014 to June 2023. Each field day, we recorded information on demographic changes (i.e., births, deaths, and dispersal), conducted a census of individuals seen and parties present, and recorded behavioral observations of adults and subadults using 20‐min focal follows (Altmann 1974). We recorded behavioral observations on electronic forms using the Pendragon software (Pendragon Software Corporation, USA), which ran on cellular phones (Samsung Note 2 and Gigaset GX290). On average, we had 6 focal follows per individual per month, evenly distributed across different times of day. During focal follows, we continuously recorded the focal animal's activity (i.e., moving, feeding, resting, and socializing), as well as all social behaviors, such as grooming and sitting in contact (Dal Pesco and Fischer 2025). We monitored daily female–male associations by observing interactions between females and males (i.e., frequency of copulations, grooming bouts, contact‐sit bouts, greetings, aggression, and duration of grooming and contact‐sit bouts) (Dal Pesco and Fischer 2018; Dal Pesco et al. 2021). We used these behaviors to monitor associations based on previous findings that female Guinea baboons' primary social partner and nearly their only mating partner is the male of their unit (Goffe et al. 2016).

### 2.3 Meat‐Eating Event Data Collection

We recorded meat‐eating events opportunistically with photographs or video recordings from video cameras (handheld Panasonic HC‐X909 and GoPro Hero8) in combination with written descriptions in electronic forms using the software Pendragon. In the case of video recordings, we verbally described the scene as it unfolded, including the identities of all adults or subadults that initially caught the prey or subsequently were transferred part or all of the carcass.

### 2.4 Data Analysis

#### 2.4.1 Male Meat Acquisition and Number of Associated Females

To test our hypothesis that “males who acquire meat more often have more females in their units”, we used a generalized linear mixed model (GLMM) with a Poisson error distribution in which the response was the mode number of females associated with a male for each year, fit with the R package lme4 version 1.1‐21 (Bates et al. 2015). The model included two predictors measuring males' meat‐acquisition abilities: “prey caught” and “meat acquisition.” Prey caught was the number of times a male caught prey each year. We included this term to test whether females prefer males who actually found and captured prey. We defined meat acquisition as the total number of times a male possessed a carcass in a given year. This term was a more general measure of a male's motivation and ability to obtain meat, whether by capturing prey or acquiring it from others. The number of prey caught and meat acquired was divided by the number of meat‐eating events in each male's party in the given year to account for differences in the relative availability of prey/meat. Thus, the predictors can be understood as the number of times males caught prey or acquired a carcass, given the number of opportunities to do so each year. The two predictors were *z*‐transformed to ease model convergence and interpretation (Schielzeth 2010). We also included a categorical variable with four levels as a fixed effect in the model that was each male's most common age category for the given year (mode male age) to account for the known fact that prime‐aged males have larger numbers of associated females (for age category definitions see CRP Simenti work manual; Dal Pesco and Fischer 2025). We included “male‐ID” and “party‐ID” as random intercepts to account for repeated observations of the same individuals and parties. Finally, we included random slopes for prey caught and meat acquired within male‐ and party‐IDs into the model (Barr et al. 2013; Forstmeier and Schielzeth 2011).

#### 2.4.2 Male Meat Acquisition and Female Tenure

To test our hypothesis that “females stay longer in the units of males who transfer meat to them more often,” we used a survival model (Cox proportional hazard model) fit with coxme version 2.2‐20 (Therneau 2018). We chose this approach because it allowed us to include all male–female associations and to censor those that were still ongoing. Additionally, survival models test how the “time until an event” (i.e., the end of a relationship) is affected by the outcome of one or more variables (i.e., frequency of meat transfers), which allowed us to ask a slightly more causal form of our question: “Is the amount of time until a female leaves her primary male affected by the frequency with which he acquires meat or transfers meat to her?”. The response in the model was the duration of each female–male association, from start to finish, measured in months, with an indication of whether the association was ongoing (i.e., right‐censored). Some female–male dyads were already associated when data collection began in 2014 (i.e., left‐censored); therefore, we regarded all associations as “minimum unit tenures,” as we did not know the extent of these associations before 2014. The main predictors were “meat possessions,” that is, the frequency with which each primary male possessed a carcass, and “meat transferred,” that is, the frequency with which they transferred meat to the female during their association. To account for observation effort, we calculated both frequencies by dividing counts of meat possession and transfers by the number of days the male was observed during the male–female association. We *z*‐transformed possession and transfer frequencies to ease model convergence (Schielzeth 2010). We also included whether or not a male was prime‐aged (yes, no; for age categories see Simenti work manual: Dal Pesco and Fischer 2025) as a fixed effect to control for prime‐aged males' tendency to have larger units. We used a binary measure of male age to facilitate model convergence: the dataset contained very few data points in certain age categories, which would have made it difficult for the model to estimate the overall effect of age. Similarly, we included “mode female age” in the model, as older females would necessarily have had the opportunity to accumulate longer unit tenures than younger females. We also included “female‐ID” and “male‐ID” as random intercepts to account for repeated observations of the same individuals in different dyads. Random slopes were not included because they were not theoretically identifiable for any of the predictor‐grouping pairs (Barr et al. 2013; Forstmeier and Schielzeth 2011).

#### 2.4.3 Model Validation

Before fitting our models, we inspected all quantitative predictors for roughly symmetric distributions. After fitting the models as an overall test of our main predictors and to avoid “cryptic multiple testing,” we conducted full‐null model comparisons (Forstmeier and Schielzeth 2011), wherein the null model lacked the primary predictors in the fixed effects part but was otherwise identical to the full model. The comparison was based on a likelihood ratio test (Dobson 2002). After fitting the Poisson model, we checked the mean–variance assumption by examining the dispersion parameter, which did not deviate much from one (dispersion parameter = 0.621). We determined variance inflation factors (VIFs) using the VIF function of the car package (version 3.0–3). Assessment of VIFs did not reveal any collinearity issues (max VIF = 1.25) (Quinn and Keough 2023). We assessed model stability at the level of the estimated coefficients by excluding levels of the random‐effect factors one at a time using a function written by R.M. (Nieuwenhuis 2012). The models were all found to be suitably stable. For the Poisson model, we determined 95% confidence intervals for model estimates and fitted values using a parametric bootstrap (*N* = 1000 bootstraps) with the function bootMer of the lme4 package. We fitted the models in R (version 4.2.0; R Core Team 2022).

## 3 Results

**FIGURE 1:**
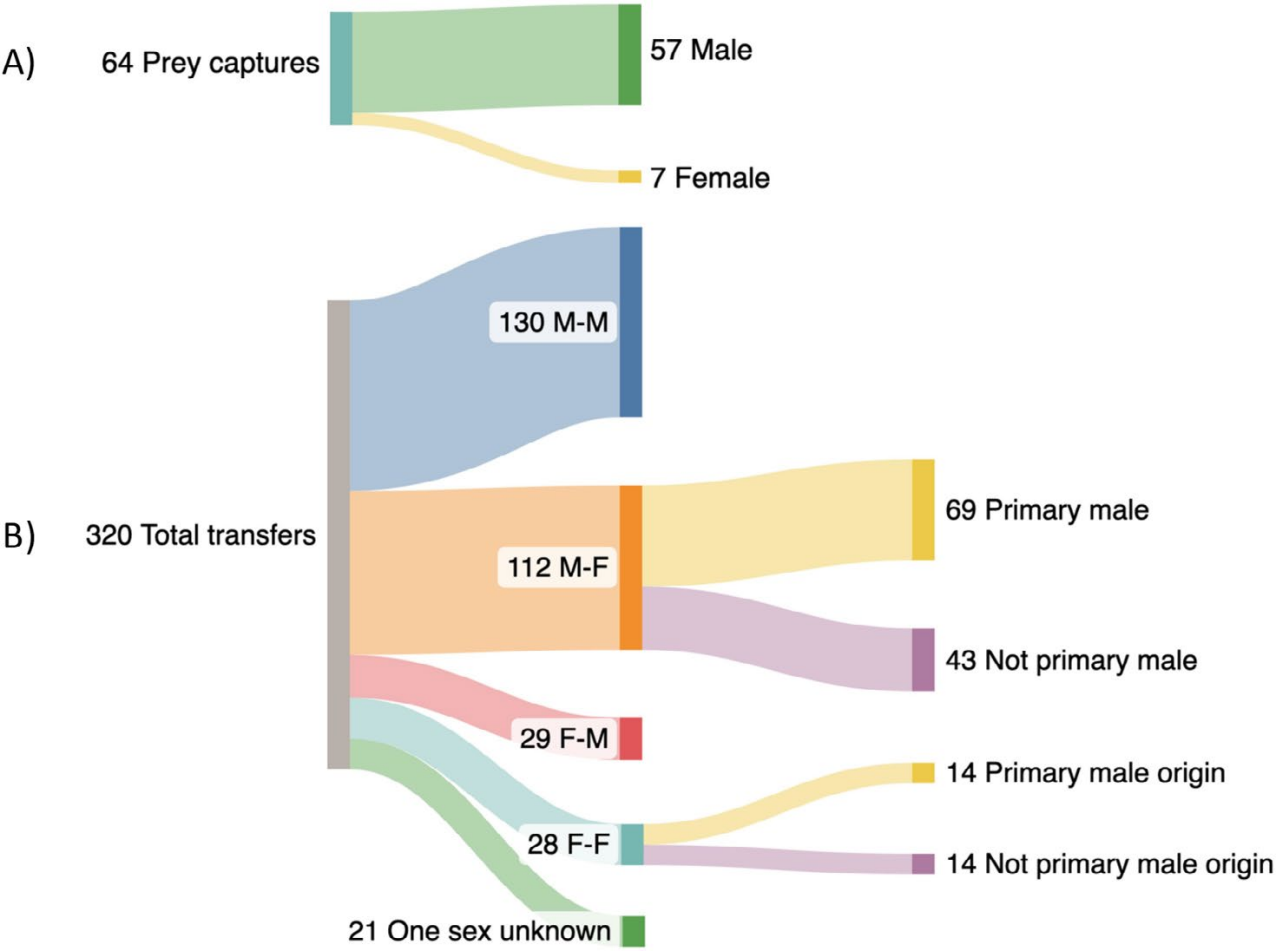
Shows two Sankey diagrams of (A) prey capture events split by the sex of individual that initially caught the prey, and (B) meat transfers split by the sex of possessor and recipient, and in the case of female recipients, whether the meat originated from her primary male.

From April 2014 to June 2023, we recorded 109 meat‐eating events at a rate of approximately 1.12 per month or 2.79 events per 100 h of observation. The events comprised 320 meat transfers, in which 111 individuals from 13 parties participated as either possessors or recipients. In 57 of the 64 events in which the prey‐catcher was identified, the prey was caught by males (Figure 1A). In the remaining seven events (11%), the prey was caught by females. This is the first reported observation of female prey capture in this species. Females were recipients in 140/320 transfers, 69 of which came from their primary male, and 14 others came from females in their unit originating with prey caught by the primary male (Figure 1B). Thus, a large share of the meat that females accessed came from their primary male (83/140; 59%). Of the remaining 57 transfers (41%) that females received, 43 came from males other than their primary male, and 14 came from females whose meat originated from outside their unit (Figure 1B).

### 3.1 Male Meat Acquisition and Number of Associated Females

**FIGURE 2:**
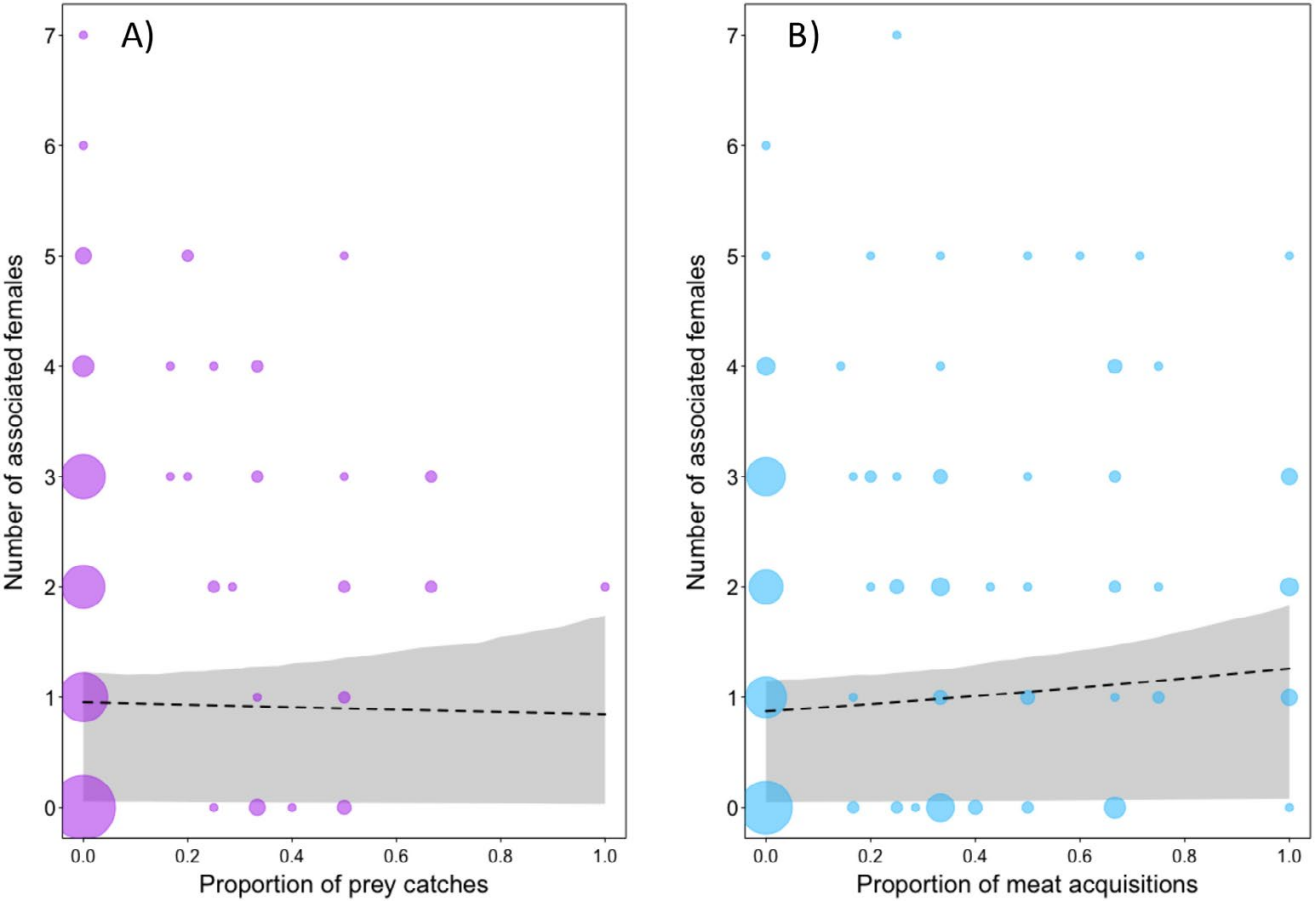
Relationship between the number of associated females (mode per year) and (A) the proportion of times males captured the prey item or (B) acquired meat from all the meat‐eating events in their party each year. There was no evidence of a relationship between prey captures (GLMM: *n* = 235, *p* = 0.747) or meat acquisition (GLMM: *n* = 235, *p* = 0.129) and the number of associated females. Points represent the males in a given year (2014–2023), and the area of the points shows the number of males that have the same yearly value (range: A: 1–64 and B: 1–43). The dashed line depicts the fitted model and the gray polygon with the bootstrapped 95% confidence intervals, with all other predictors being centered.

The model assessing the link between male meat acquisition and number of associated females included 235 observations from 57 males across eight parties, with a median number of associated females of 1 (range: 0–7). The number of males that caught prey at least once was 19 (range: 1–3 times per year), and the number that acquired meat at least once was 34 (range: 1–8 times per year), meaning 15 of the 34 males that acquired meat did not catch it themselves. The full‐null model comparison was not significant (*χ* ^2^ = 2.41, df = 2, *p* = 0.299). Thus, we found no significant relationship between the number of associated females in a male's unit and the number of times a male caught prey (Estimate = −0.232, SE = 0.072, *p* = 0.747; Table 1) or possessed a carcass (Estimate = 0.113, SE = 0.074, *p* = 0.129; Table 1). In other words, males who caught prey (Figure 2A) or acquired meat more often (Figure 2B) did not have more associated females than males who caught prey or acquired meat less often. The largest effect size among the model's factors was male age: early‐ and mid‐prime males had more associated females in their units than subadult, late‐prime, and old males (Table 1).

**TABLE 1:** GLMM model output of the number of females associated with a male predicted by meat possession and prey capture frequency.

Term	Estimate ± SE	Lower CI	Upper CI	*χ* ^2^	df	*p*	Min	Max
Intercept	0.516 ± 0.150	0.2313	0.787				0.417	0.603
Meat possessions^a^	0.113 ± 0.074	−0.447	0.249	2.135	1	0.129	0.087	0.139
Prey captures^b^	−0.232 ± 0.072	−0.186	0.111	0.078	1	0.747	−0.049	0.022
Mode male age LPAM^c^	−0.743 ± 0.264	−1.331	−0.302	63.454^d^	4^d^	< 0.001^d^	−1.046	−0.462
Mode male age LSAM^c^	−1.378 ± 0.315	−2.092	−0.859				−1.835	−0.970
Mode male age MPAM^c^	0.003 ± 0.144	−0.260	0.257				−0.049	0.108
Mode male age OAM^c^	−3.500 ± 1.019	−36.337	−2.183				−40.797	−2.804

^a^ Meat possessions were *z*‐transformed to a mean of 0 and a standard deviation of 1; the original mean was 0.906, and the standard deviation was 1.746.

^b^ Prey captures were *z*‐transformed to a mean of 0 and a standard deviation of 1; the original mean was 0.195, and the standard deviation was 0.518.

^c^ The reference level for this variable was early prime adult male (EPAM). The other levels are large subadult males (LSAMs), mid‐prime adult males (MPAMs), late‐prime adult males (LPAMs), and old adult males (OAMs).

^d^ The indicated test is for the overall effect of age.

### 3.2 Male Meat Acquisition and Female Tenure

**FIGURE 3:**
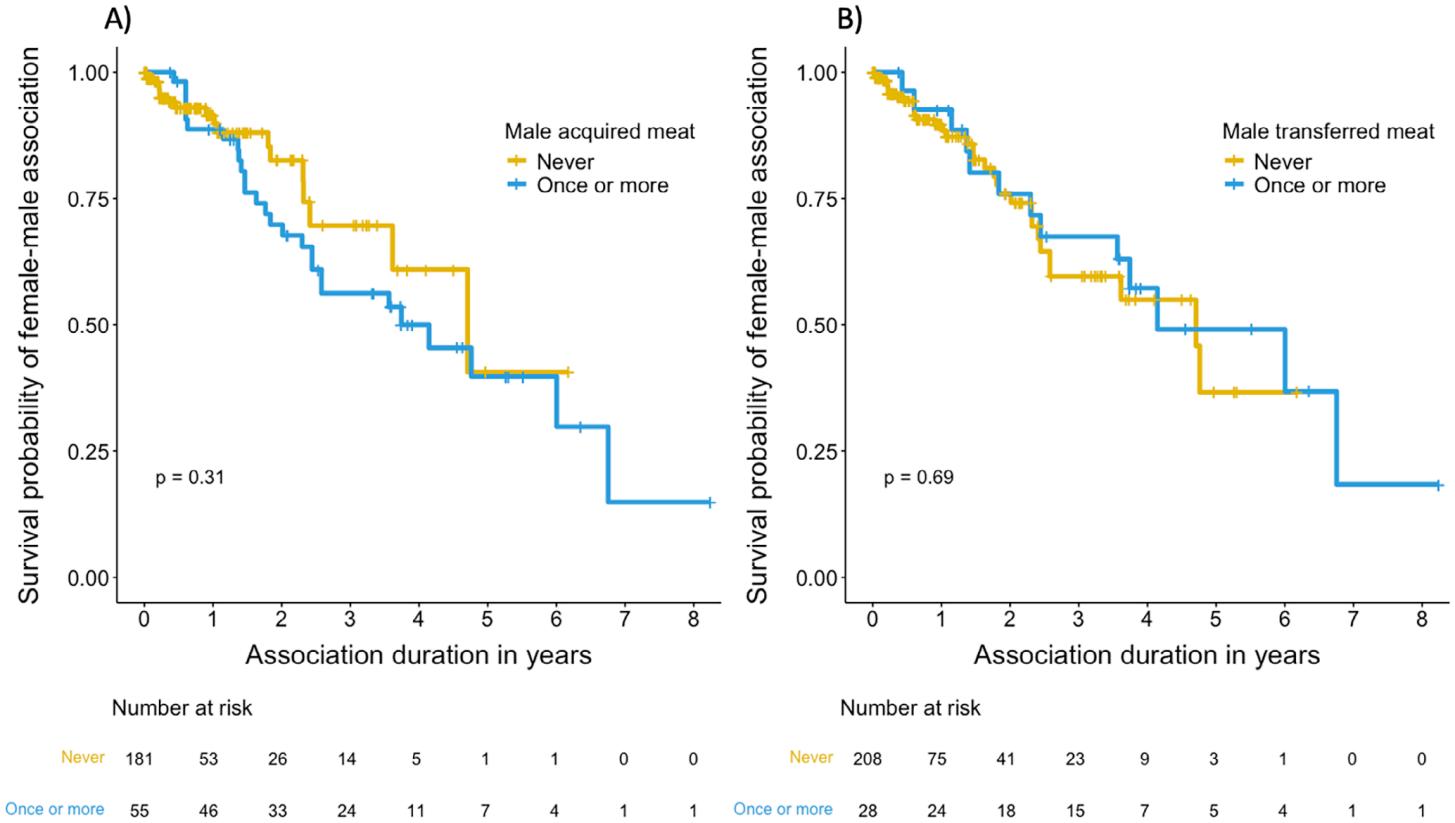
Kaplan–Meier survival curves for the effects of males (A) acquiring meat and (B) transferring meat to their female on the “survival” of the male–female association. Separate lines are for males who never acquired or transferred meat (blue) and males who acquired or transferred meat at least once (yellow). This figure is simplified to illustrate the result. In the model, meat acquisition and transfer were continuous rather than binary variables. Risk tables beneath the plots give the number of female–male associations that “survived” to each yearly increment.

The model examining the link between male meat acquisition, meat transfer, and female tenure comprised 236 dyads, formed by 99 females and 54 males, with a median association duration of 269 days (range: 1–2967 days). Of those dyads, there were 55 in which the male acquired meat at least once (range: 1–12) and 28 dyads in which males transferred meat at least once (range: 1–7). The full‐null model comparison was not significant (*χ* ^2^ = 3.08, df = 2, *p* = 0.21). Consequently, we found no significant relationship between the duration of a female's tenure and the number of times her male acquired meat (Estimate = −0.103, SE = 0.186, *p* = 0.577; Table 2; Figure 3A) or transferred meat to her (Estimate = −0.157, SE = 0.195, *p* = 0.419; Table 2; Figure 3B). As in the number of associated females model above, we found that prime‐aged males were more successful at maintaining a unit, as females associated with them for longer than with non‐prime‐aged males (Estimate = 2.971, standard error = 1.152).

**TABLE 2:** Survival model output for female unit tenure predicted by male meat possession and transfer frequency.

Term	Estimate ± SE	*χ* ^2^	df	*p*	Min	Max
Male transfer events^a^	−0.154 ± 0.192	0.236	1	0.626	−0.291	0.071
Male meat possessions^b^	−0.089 ± 0.177	5.011	1	0.251	−0.226	0.040
Mode male age NP^c^	2.975 ± 1.150	5.223	1	0.009	2.516	3.675
Mode female age OAF^d^	0.765 ± 1.304	6.645^e^	3^e^	0.156^e^	−22.851	1.243
Mode female age SAF^d^	1.145 ± 0.504				0.992	1.352
Mode female age YAF^d^	0.562 ± 0.534				0.341	0.760

^a^ Meat transfer events were *z*‐transformed to a mean of 0 and a standard deviation of 1; the original mean was 0.207, and the standard deviation was 0.779.

^b^ Meat possessions were *z*‐transformed to a mean of 0 and a standard deviation of 1; the original mean was 0.648, and the standard deviation was 1.68.

^c^ NP refers to non‐prime aged. The reference level for this variable is prime‐aged.

^d^ The reference level for this variable is mature adult females (MAFs). The other levels are subadult females (SAFs), young adult females (YAFs), and old adult females (OAFs). The indicated significance test is for the overall effect of male age.

^e^ The indicated test is for the overall effect of age.

## 4 Discussion

We found no evidence that female Guinea baboons preferred to join or remain in the units of males who acquired or transferred meat more often. Thus, prey capturing and meat transfers are unlikely to be signals of male quality in Guinea baboons. Although females have been shown to increase their affiliative interactions with their primary males to access desirable food in their possession (O'Hearn, Beckmann, et al. 2025), they do not appear to use males' meat acquisition or transfer skills to choose mates. Thus, contrary to our predictions, we found that male meat acquisition was unlikely to be relevant to female reproductive decisions.

The simplest explanation for female Guinea baboons not preferring males based on their record of acquiring meat is that meat‐eating events are relatively infrequent in the species. Rates of prey capture in Guinea baboons (2.79 prey per 100 h, 1.12 prey per month) are comparable to other species of baboons ( *Papio ursinus* , 1.27 prey event per 100 h; *Papio hamadryas* : 0.1–2.8 prey per 100 h; *Papio cynocephalus* : 1.78 prey per 100 h; *Papio anubis* : 4.54 prey per 100 h, Bugir et al. 2021; Hamilton III and Busse 1978; Hausfater 1976; Kummer 1968; Schreier et al. 2019; Swedell 2006; Watts 2020), and capuchins ( *Cebus capucinus* : 5.35 prey per 100 h; *Sapajus libidinosus* : 0.3 prey per 100 h, Watts 2020). However, prey capture rates are generally lower than capture rates of chimpanzees (Kanyawara–Ngogo: 0.8–7.6 per month, Watts 2020) and far below the weekly hunts of many human hunter‐gatherer societies (Hill et al. 1993; Kishigami 2000; Naylor et al. 2021). The infrequent occurrence of meat‐eating events could mean that, despite the dietary value of vertebrate prey and its value as an indicator of co‐feeding tolerance, a male's ability to acquire and transfer meat has insufficient impact on his females' fitness to merit their use as a mate choice criterion. It could be that meat access has less impact than other as‐yet‐untested traits that females might use as criteria when choosing a primary male. Females could be attentive to physical signals of male quality such as female geladas' ( *Theropithecus gelada* ) preference for males with redder patches of skin on their chest—a signal associated with higher social status and membership of “better” groups (Bergman et al. 2009). Similarly, females could prefer males with more dissimilar major histocompatibility complex (MHC) genotypes to themselves, as has been studied in the multilevel society of golden snub‐nosed monkeys (*Rhinopithecus roxellana*) (Guo et al. 2010; Zhang et al. 2023). Females could also attend to male behavioral tendencies such as their willingness to care for offspring (Rosenbaum et al. 2018), degree of tolerance toward unit females (Goffe et al. 2016), or general attentiveness to social or external threats may outweigh the impact of meat provisioning, as they more consistently and directly impact females' reproductive output and safety. Additionally, other features of the unit, such as the number and identities of other females and their offspring, may also influence female unit choice and tenure. Females may avoid units with antagonistic females or prefer those with existing affiliative partners, as seen in female mountain gorillas ( *Gorilla beringei beringei* ) who preferred to join males' harems when they contained familiar females (Martignac et al. 2025).

Another reason male meat acquisition may not affect female partner preference is that females have more pathways to access meat than previously reported (Goffe and Fischer 2016). In our expanded dataset covering 9 years of meat‐eating events, primary males were indeed the main source of meat for their unit females (59%). However, nearly half of the meat consumed by females came from sources other than their primary male (Figure 1). Additionally, we report the first observations of female prey capture in this species (11% of captures), consistent with evidence of prey capture by both sexes in other baboon species (Allan et al. 2022; Schreier et al. 2019; Strum 1975). The prey items and capture methods of females in this sample did not deviate from those of males, nor were there consistent characteristics of age, parity, reproductive status, party, or unit among female prey capturers to indicate they are aberrant or part of a particular demographic subset. Together, these findings indicate that female Guinea baboons are less dependent on males for access to meat than previously thought, which could further explain the lack of mate preference for males who acquire more meat.

In conclusion, acquiring and transferring meat did not appear to function as a signal of male quality in Guinea baboons. This finding is consistent with the wider pattern in primates that male‐to‐female meat transfers are not clearly associated with increased reproductive opportunities for males (Jaeggi and Gurven 2013; Jaeggi and Van Schaik 2011; but see Gomes and Boesch 2009). In fact, in our sample, most transfers occurred between males, which may reflect the strength of male–male relationships in Guinea baboons (O'Hearn, Beckmann, et al. 2025) and the central, tolerant role they play in their society (Dal Pesco et al. 2021). It is also consistent with evidence from feeding experiments in which high‐quality food sources produced by males were often fed on by other males (O'Hearn et al. 2024; O'Hearn, Beckmann, et al. 2025). Our results thus underscore the differences between the roles of meat transfers in human foraging groups and some other nonhuman primate species (Gurven 2004; Kaplan and Gurven 2005). Despite the widespread transfers of meat in Guinea baboons, which look superficially similar to patterns in humans (O'Hearn, Beckmann, et al. 2025) and the shared evolutionary past both behaviors stem from (Wood and Gilby 2018), meat transfers in Guinea baboons do not play a central role as meat sharing does in human hunter‐gatherer societies. At the same time, our findings raise important questions specific to Guinea baboons. If meat acquisition and transfer are not central to male quality assessment, what criteria do females rely on when choosing males? It could be that females prefer males based on the quality of their parental care or the identities of females already in their units. Similarly, if the capacity to observe and evaluate others' foraging skills did not evolve in this system to inform mate choice, then what selective pressure brought it about? It could be that evaluating others' foraging skills is useful for identifying social learning models, or skillful manipulative foraging could be correlated with other fitness‐relevant behavioral traits like strength or dexterity. Both possibilities are worth exploring as future research questions.

## Author Contributions

**Federica Dal Pesco:** methodology, data curation, writing – review and editing. **Julia Fischer:** supervision, resources, project administration, writing – review and editing, conceptualization, funding acquisition. **Roger Mundry:** methodology, formal analysis, writing – review and editing. **William J O'Hearn:** conceptualization, methodology, investigation, formal analysis, funding acquisition, writing – original draft, writing – review and editing.

## Funding

This research was funded by the Deutsche Forschungsgemeinschaft (DFG, German Research Foundation) under Project‐ID 454648639 SFB 1528 “Cognition of Interaction” and Project‐ID 254142454/GRK 2070 “Understanding Social Relationships.” Support by the Leibniz ScienceCampus “Primate Cognition” (Audacity Fund AF2020_03) is gratefully acknowledged.

## Ethics Statement

This research was conducted within the regulations set by Senegalese authorities and the guidelines for the ethical treatment of nonhuman animals set down by the Association for the Study of Animal Behaviour (ASAB Ethical Committee/ABS Animal Care Committee 2023).

## Conflicts of Interest

The authors declare no conflicts of interest.

## Data Availability

The data that support the findings of this study are openly available in Data for “Meat acquisition skill and male reproductive success in Guinea baboons” at https://osf.io/9thk4/overview.

## References

[ajpa70327-bib-0001] Achorn, A. , S. Lindshield , P. I. Ndiaye , J. Winking , and J. D. Pruetz . 2023. “Reciprocity and Beyond: Explaining Meat Transfers in Savanna‐Dwelling Chimpanzees at Fongoli, Senegal.” American Journal of Biological Anthropology 182, no. 2: 224–236. 10.1002/ajpa.24815. 37452552

[ajpa70327-bib-0002] Albo, M. J. , and F. G. Costa . 2010. “Nuptial Gift‐Giving Behaviour and Male Mating Effort in the Neotropical Spider *Paratrechalea ornata* (Trechaleidae).” Animal Behaviour 79, no. 5: 1031–1036. 10.1016/j.anbehav.2010.01.018.

[ajpa70327-bib-0003] Allan, A. T. L. , L. R. LaBarge , C. Howlett , et al. 2022. “Patterns of Predation and Meat‐Eating by Chacma Baboons in an Afromontane Environment.” Folia Primatologica 94, no. 1: 13–36. 10.1163/14219980-bja10004.

[ajpa70327-bib-0004] Altmann, J. 1974. “Observational Study of Behavior: Sampling Methods.” Behaviour 49, no. 3–4: 227–266. 10.1163/156853974X00534. 4597405

[ajpa70327-bib-0005] Alvard, M. S. , and A. Gillespie . 2004. “Good Lamalera Whale Hunters Accrue Reproductive Benefits.” In Research in Economic Anthropology, vol. 23, edited by M. Alvard, 225–247. Emerald (MCB UP). 10.1016/S0190-1281(04)23009-8.

[ajpa70327-bib-0006] Amat, J. A. 2000. “Courtship Feeding, Food Sharing, or Tolerated Food Theft Among Paired Red‐Crested Pochards ( *Netta rufina* )?” Journal für Ornithologie 141, no. 3: 327. 10.1046/j.1439-0361.2000.00029.x.

[ajpa70327-bib-0007] Anderson, J. R. , and W. C. McGrew . 1984. “Guinea Baboons ( *Papio papio* ) at a Sleeping Site.” American Journal of Primatology 6, no. 1: 1–14. 10.1002/ajp.1350060102. 31986846

[ajpa70327-bib-0008] ASAB Ethical Committee/ABS Animal Care Committee . 2023. “Guidelines for the Ethical Treatment of Nonhuman Animals in Behavioural Research and Teaching.” Animal Behaviour 195: I–XI. 10.1016/j.anbehav.2022.09.006.

[ajpa70327-bib-0010] Barr, D. J. , R. Levy , C. Scheepers , and H. J. Tily . 2013. “Random Effects Structure for Confirmatory Hypothesis Testing: Keep It Maximal.” Journal of Memory and Language 68, no. 3: 255–278. 10.1016/j.jml.2012.11.001. PMC3881361 24403724

[ajpa70327-bib-0011] Bates, D. , M. Maechler , B. Bolker , and S. Walker . 2015. “Fitting Linear Mixed‐Effects Models Using lme4.” Journal of Statistical Software 67, no. 1: 1–48. 10.18637/jss.v067.i01.

[ajpa70327-bib-0012] Bergman, T. J. , L. Ho , and J. C. Beehner . 2009. “Chest Color and Social Status in Male Geladas ( *Theropithecus gelada* ).” International Journal of Primatology 30, no. 6: 791–806. 10.1007/s10764-009-9374-x.

[ajpa70327-bib-0013] Boesch, C. , and H. Boesch . 1989. “Hunting Behavior of Wild Chimpanzees in the Taï National Park.” American Journal of Physical Anthropology 78, no. 4: 547–573. 10.1002/ajpa.1330780410. 2540662

[ajpa70327-bib-0014] Boese, G. K. 1975. “Social Behavior and Ecological Considerations of West African Baboons (Papio Papio).” In Socioecology and Psychology of Primates, edited by R. H. Tuttle , 205–230. Mouton Publisher.

[ajpa70327-bib-0015] Bugir, C. K. , C. A. Peres , K. S. White , et al. 2021. “Prey Preferences of Modern Human Hunter‐Gatherers.” Food Webs 26: e00183. 10.1016/j.fooweb.2020.e00183.

[ajpa70327-bib-0016] Chapais, B. 2009. Primeval Kinship: How Pair‐Bonding Gave Birth to Human Society. Harvard University Press.

[ajpa70327-bib-0017] Codron, D. , J. A. Lee‐Thorp , M. Sponheimer , D. de Ruiter , and J. Codron . 2008. “What Insights Can Baboon Feeding Ecology Provide for Early Hominin Niche Differentiation?” International Journal of Primatology 29, no. 3: 757–772. 10.1007/s10764-008-9261-x.

[ajpa70327-bib-0018] Dal Pesco, F. , and J. Fischer . 2018. “Greetings in Male Guinea Baboons and the Function of Rituals in Complex Social Groups.” Journal of Human Evolution 125: 87–98. 10.1016/j.jhevol.2018.10.007. 30502900

[ajpa70327-bib-0019] Dal Pesco, F. , and J. Fischer . 2025. “CRP Simenti Work Manual and Guides.” https://osf.io/ak3w4/.

[ajpa70327-bib-0020] Dal Pesco, F. , F. Trede , D. Zinner , and J. Fischer . 2021. “Kin Bias and Male Pair‐Bond Status Shape Male‐Male Relationships in a Multilevel Primate Society.” Behavioral Ecology and Sociobiology 75, no. 24: 1–14. 10.1007/s00265-020-02960-8.

[ajpa70327-bib-0021] Dal Pesco, F. , F. Trede , D. Zinner , and J. Fischer . 2022. “Male–Male Social Bonding, Coalitionary Support and Reproductive Success in Wild Guinea Baboons.” Proceedings in the Royal Society B 289, no. 1975: 20220347. 10.1098/rspb.2022.0347. PMC9130795 35611539

[ajpa70327-bib-0022] de Kort, S. R. , N. J. Emery , and N. S. Clayton . 2006. “Food Sharing in Jackdaws, *Corvus monedula* : What, Why and With Whom?” Animal Behaviour 72, no. 2: 297–304. 10.1016/j.anbehav.2005.10.016.

[ajpa70327-bib-0023] DeVore, I. , and S. L. Washburn . 1963. “Baboon Ecology and Human Evolution.” In African Ecology and Human Evolution, edited by F. C. Howell and F. Bourlière , 335–367. Routledge.

[ajpa70327-bib-0024] Dobson, A. 2002. An Introduction to Generalized Linear Models. Chapman & Hall/CRC.

[ajpa70327-bib-0025] Emery, N. , S. De Kort , A. Von Bayern , and N. Clayton . 2007. “The Role of Food‐ and Object‐Sharing in the Development of Social Bonds in Juvenile Jackdaws ( *Corvus monedula* ).” Behaviour 144, no. 6: 711–733. 10.1163/156853907781347826.

[ajpa70327-bib-0026] Fischer, J. , J. P. Higham , S. C. Alberts , et al. 2019. “Insights Into the Evolution of Social Systems and Species From Baboon Studies.” eLife 8: e50989. 10.7554/eLife.50989. 31711570 PMC6850771

[ajpa70327-bib-0027] Fischer, J. , G. H. Kopp , F. Dal Pesco , et al. 2017. “Charting the Neglected West: The Social System of Guinea Baboons.” American Journal of Physical Anthropology 162: 15–31. 10.1002/ajpa.23144. 28105722 PMC6586040

[ajpa70327-bib-0028] Forstmeier, W. , and H. Schielzeth . 2011. “Cryptic Multiple Hypotheses Testing in Linear Models: Overestimated Effect Sizes and the Winner's Curse.” Behavioral Ecology and Sociobiology 65, no. 1: 47–55. 10.1007/s00265-010-1038-5. 21297852 PMC3015194

[ajpa70327-bib-0030] Goffe, A. S. , and J. Fischer . 2016. “Meat Sharing Between Male and Female Guinea Baboons ( *Papio papio* ).” Primate Biology 3: 1–8. 10.5194/pb-3-1-2016.

[ajpa70327-bib-0031] Goffe, A. S. , D. Zinner , and J. Fischer . 2016. “Sex and Friendship in a Multilevel Society: Behavioural Patterns and Associations Between Female and Male Guinea Baboons.” Behavioral Ecology and Sociobiology 70, no. 3: 323–336. 10.1007/s00265-015-2050-6. 26900211 PMC4748025

[ajpa70327-bib-0032] Gomes, C. M. , and C. Boesch . 2009. “Wild Chimpanzees Exchange Meat for Sex on a Long‐Term Basis.” PLoS One 4, no. 4: e5116. 10.1371/journal.pone.0005116. 19352509 PMC2663035

[ajpa70327-bib-0033] González‐Solís, J. , E. Sokolov , and P. H. Becker . 2001. “Courtship Feedings, Copulations and Paternity in Common Terns, *Sterna hirundo* .” Animal Behaviour 61, no. 6: 1125–1132. 10.1006/anbe.2001.1711.

[ajpa70327-bib-0034] Grueter, C. C. , X. Qi , D. Zinner , et al. 2020. “Multilevel Organisation of Animal Sociality.” Trends in Ecology & Evolution 35, no. 9: 834–847. 10.1016/j.tree.2020.05.003. 32473744

[ajpa70327-bib-0035] Guo, S. , W. Ji , M. Li , H. Chang , and B. Li . 2010. “The Mating System of the Sichuan Snub‐Nosed Monkey ( *Rhinopithecus roxellana* ).” American Journal of Primatology 72, no. 1: 25–32. 10.1002/ajp.20747. 19768744

[ajpa70327-bib-0036] Gurven, M. 2004. “To Give and to Give Not: The Behavioral Ecology of Human Food Transfers.” Behavioral and Brain Sciences 27, no. 4: 543–559. 10.1017/S0140525X04000123.

[ajpa70327-bib-0037] Gurven, M. , and C. Von Rueden . 2006. “Hunting, Social Status and Biological Fitness.” Biodemography and Social Biology 53, no. 1–2: 81–99. 10.1080/19485565.2006.9989118. 21516952

[ajpa70327-bib-0038] Hamilton, W. J., III , and C. D. Busse . 1978. “Primate Carnivory and Its Significance to Human Diets.” Bioscience 28, no. 12: 761–766. 10.2307/1307249.

[ajpa70327-bib-0039] Harding, R. S. O. 1975. “Meat‐Eating and Hunting in Baboons.” In Socioecology and Psychology of Primates, edited by R. H. Tuttle , 245–257. Mouton Publisher.

[ajpa70327-bib-0040] Hausfater, G. 1976. “Predatory Behaviour of Yellow Baboons.” Behaviour 56: 44–68.

[ajpa70327-bib-0041] Hawkes, K. , J. F. O'Connell , and N. G. Jones . 1991. “Hunting Income Patterns Among the Hadza: Big Game, Common Goods, Foraging Goals and the Evolution of the Human Diet.” Philosophical Transactions of the Royal Society of London. Series B, Biological Sciences 334, no. 1270: 243–251. 10.1098/rstb.1991.0113. 1685582

[ajpa70327-bib-0042] Heinrich, B. 1988. “Winter Foraging at Carcasses by Three Sympatric Corvids, With Emphasis on Recruitment by the Raven, *Corvus corax* .” Behavioral Ecology and Sociobiology 23, no. 3: 141–156.

[ajpa70327-bib-0043] Hill, K. , H. Kaplan , and K. Hawkes . 1993. “On Why Male Foragers Hunt and Share Food.” Current Anthropology 34, no. 5: 701–710. 10.1086/204213.

[ajpa70327-bib-0044] Jaeggi, A. V. , and M. Gurven . 2013. “Natural Cooperators: Food Sharing in Humans and Other Primates.” Evolutionary Anthropology: Issues, News, and Reviews 22, no. 4: 186–195. 10.1002/evan.21364. 23943272

[ajpa70327-bib-0045] Jaeggi, A. V. , and M. Gurven . 2018. “Food‐Sharing Models.” In The International Encyclopedia of Anthropology, edited by H. Callan , 1st ed., 1–8. Wiley. 10.1002/9781118924396.wbiea1655.

[ajpa70327-bib-0046] Jaeggi, A. V. , and C. P. Van Schaik . 2011. “The Evolution of Food Sharing in Primates.” Behavioral Ecology and Sociobiology 65, no. 11: 2125–2140. 10.1007/s00265-011-1221-3.

[ajpa70327-bib-0047] Jolly, C. J. 1970. “The Seed‐Eaters: A New Model of Hominid Differentiation Based on a Baboon Analogy.” Man 5, no. 1: 5–26. 10.2307/2798801.

[ajpa70327-bib-0048] Kaplan, H. , and M. Gurven . 2005. “The Natural History of Human Food Sharing and Cooperation: A Review and a New Multi‐Individual Approach to the Negotiation of Norms.” In Moral Sentiments and Material Interests: The Foundations of Cooperation in Economic Life, edited by H. Gintis , S. Bowles , R. Boyd , and E. Fehr . MIT Press.

[ajpa70327-bib-0049] Kaplan, H. , and K. Hill . 1985. “Hunting Ability and Reproductive Success Among Male Ache Foragers: Preliminary Results.” Current Anthropology 26, no. 1: 131–133. 10.1086/203235.

[ajpa70327-bib-0050] Kishigami, N. 2000. “Contemporary Inuit Food Sharing and Hunter Support Program of Nunavik, Canada.” Senri Ethnological Studies 53: 171–192. 10.15021/00002848.

[ajpa70327-bib-0051] Kummer, H. 1968. “Social Organization of Hamadryas Baboons: A Field Study.” In Bibliotheca Primatologica. University of Chicago Press.

[ajpa70327-bib-0052] Martignac, V. , W. Eckardt , J. P. S. Mucyo , et al. 2025. “Dispersed Female Networks: Female Gorillas' Inter‐Group Relationships Influence Dispersal Decisions.” Proceedings of the Royal Society B: Biological Sciences 292, no. 2052: 20250223. 10.1098/rspb.2025.0223. PMC12324879 40763812

[ajpa70327-bib-0053] Mitani, J. C. , and D. P. Watts . 2001. “Why Do Chimpanzees Hunt and Share Meat?” Animal Behaviour 61, no. 5: 915–924. 10.1006/anbe.2000.1681.

[ajpa70327-bib-0054] Naylor, A. W. , T. Pearce , J. D. Ford , D. Fawcett , P. Collings , and S. L. Harper . 2021. “Understanding Determinants of Hunting Trip Productivity in an Arctic Community.” Frontiers in Sustainable Food Systems 5: 688350. 10.3389/fsufs.2021.688350.

[ajpa70327-bib-0083] Nieuwenhuis, R. 2012. “Influence.ME: Tools for Detecting Influential Data in Mixed Effects Models.” R Journal 4, no. 2: 38–47. 10.32614/CRAN.package.influence.ME.

[ajpa70327-bib-0056] O'Hearn, W. , L. Frank , S. Keupp , and J. Fischer . 2024. “Lessons Learned From a Cooperative Box Experiment With Wild Baboons.” Animal Behavior and Cognition 11, no. 4: 330–348. 10.26451/abc.11.04.01.2024.

[ajpa70327-bib-0057] O'Hearn, W. J. , J. Beckmann , L. Von Fersen , et al. 2025. “Increased Female Competition for Males With Enhanced Foraging Skills in Guinea Baboons.” Proceedings of the Royal Society B: Biological Sciences 292, no. 2042: 20242925. 10.1098/rspb.2024.2925. PMC11880840 40040456

[ajpa70327-bib-0058] O'Hearn, W. J. , C. Neumann , R. Mundry , F. D. Pesco , and J. Fischer . 2025. “Meat Transfer Patterns Reflect the Multi‐Level Social System of Guinea Baboons.” iScience 28, no. 11: 113619. 10.1016/j.isci.2025.113619. 41142114 PMC12547275

[ajpa70327-bib-0059] Ohrndorf, L. , R. Mundry , J. Beckmann , J. Fischer , and D. Zinner . 2025. “Impact of Food Availability and Predator Presence on Patterns of Landscape Partitioning Among Neighbouring Guinea Baboon ( *Papio papio* ) Parties.” Movement Ecology 13, no. 1: 9. 10.1186/s40462-025-00534-9. 39987137 PMC11847332

[ajpa70327-bib-0060] Patzelt, A. , G. H. Kopp , I. Ndao , U. Kalbitzer , D. Zinner , and J. Fischer . 2014. “Male Tolerance and Male–Male Bonds in a Multilevel Primate Society.” Proceedings of the National Academy of Sciences of the United States of America 111, no. 41: 14740–14745. 10.1073/pnas.1405811111. 25201960 PMC4205614

[ajpa70327-bib-0061] Perry, S. , and L. Rose . 1994. “Begging and Transfer of Coati Meat by White‐Faced Capuchin Monkeys, *Cebus capucinus* .” Primates 35, no. 4: 409–415. 10.1007/BF02381950.

[ajpa70327-bib-0062] Quinn, G. P. , and M. J. Keough . 2023. Experimental Design and Data Analysis for Biologists. 2nd ed. Cambridge University Press.

[ajpa70327-bib-0082] R Core Team . 2022. R: A Language and Environment for Statistical Computing. R Foundation for Statistical Computing. https://www.R‐project.org.

[ajpa70327-bib-0063] Rhine, R. J. , G. W. Norton , G. M. Wynn , R. D. Wynn , and H. B. Rhine . 1986. “Insect and Meat Eating Among Infant and Adult Baboons ( *Papio cynocephalus* ) of Mikumi National Park, Tanzania.” American Journal of Physical Anthropology 70, no. 1: 105–118. 10.1002/ajpa.1330700115. 3728648

[ajpa70327-bib-0064] Rosenbaum, S. , L. Vigilant , C. W. Kuzawa , and T. S. Stoinski . 2018. “Caring for Infants Is Associated With Increased Reproductive Success for Male Mountain Gorillas.” Scientific Reports 8, no. 1: 15223. 10.1038/s41598-018-33380-4. 30323256 PMC6189178

[ajpa70327-bib-0065] Schielzeth, H. 2010. “Simple Means to Improve the Interpretability of Regression Coefficients.” Methods in Ecology and Evolution 1, no. 2: 103–113. 10.1111/j.2041-210X.2010.00012.x.

[ajpa70327-bib-0066] Schreier, A. L. , R. M. Schlaht , and L. Swedell . 2019. “Meat Eating in Wild Hamadryas Baboons: Opportunistic Trade‐Offs Between Insects and Vertebrates.” American Journal of Primatology 81, no. 7: e23029. 10.1002/ajp.23029. 31297842

[ajpa70327-bib-0067] Sharman, J. 1982. Feeding, Ranging and Social Organisation of the Guinea Baboon. University of St. Andrews.

[ajpa70327-bib-0068] Smith, E. A. 2004. “Why Do Good Hunters Have Higher Reproductive Success?” Human Nature 15, no. 4: 343–364. 10.1007/s12110-004-1013-9. 26189411

[ajpa70327-bib-0069] Stanford, C. B. 1998. Chimpanzee and Red Colobus: The Ecology of Predator and Prey. Harvard University Press.

[ajpa70327-bib-0070] Stevens, J. R. , and I. C. Gilby . 2004. “A Conceptual Framework for Nonkin Food Sharing: Timing and Currency of Benefits.” Animal Behaviour 67, no. 4: 603–614. 10.1016/j.anbehav.2003.04.012.

[ajpa70327-bib-0071] Strum, S. C. 1975. “Primate Predation: Interim Report on the Development of a Tradition in a Troop of Olive Baboons.” Science 187, no. 4178: 755–757. 10.1126/science.187.4178.755. 17795248

[ajpa70327-bib-0072] Swedell, L. 2006. Strategies of Sex and Survival in Hamadryas Baboons: Through a Female Lens. Prentice Hall College Division.

[ajpa70327-bib-0073] Tennie, C. , I. C. Gilby , and R. Mundry . 2009. “The Meat‐Scrap Hypothesis: Small Quantities of Meat May Promote Cooperative Hunting in Wild Chimpanzees ( *Pan troglodytes* ).” Behavioral Ecology and Sociobiology 63, no. 3: 421–431. 10.1007/s00265-008-0676-3.

[ajpa70327-bib-0074] Therneau, T. M. 2018. “coxme: Mixed Effects Cox Models.”

[ajpa70327-bib-0075] Vahed, K. 1998. “The Function of Nuptial Feeding in Insects: A Review of Empirical Studies.” Biological Reviews 73, no. 1: 43–78. 10.1111/j.1469-185X.1997.tb00025.x.

[ajpa70327-bib-0076] Watts, D. P. 2020. “Meat Eating by Nonhuman Primates: A Review and Synthesis.” Journal of Human Evolution 149: 102882. 10.1016/j.jhevol.2020.102882. 33137551

[ajpa70327-bib-0077] Wiessner, P. 1996. “Leveling the Hunter: Constraints on the Status Quest in Foraging Societies.” In Food and the Status Quest, edited by P. Wiessner and W. Schienfenhövel . Berghahn Books.

[ajpa70327-bib-0078] Wood, B. M. , and I. C. Gilby . 2018. “10. From Pan to Man the Hunter: Hunting and Meat Sharing by Chimpanzees, Humans, and Our Common Ancestor.” In Chimpanzees and Human Evolution, edited by M. N. Muller , R. W. Wrangham , and D. R. Pilbeam , 339–382. Harvard University Press. 10.4159/9780674982642-010.

[ajpa70327-bib-0079] Wood, B. M. , and F. W. Marlowe . 2013. “Household and Kin Provisioning by Hadza Men.” Human Nature 24, no. 3: 280–317. 10.1007/s12110-013-9173-0. 23813245

[ajpa70327-bib-0080] Zhang, P. , B. Zhang , D. W. Dunn , et al. 2023. “Social and Paternal Female Choice for Male MHC Genes in Golden Snub‐Nosed Monkeys ( *Rhinopithecus roxellana* ).” Molecular Ecology 32, no. 12: 3239–3256. 10.1111/mec.16932. 36942819

[ajpa70327-bib-0081] Zinner, D. , M. Klapproth , A. Schell , et al. 2021. “Comparative Ecology of Guinea Baboons ( *Papio papio* ).” Primate Biology 8, no. 1: 19–35. 10.5194/pb-8-19-2021. 34109265 PMC8182668

